# Patient-derived xenografts faithfully replicated clinical outcome in a phase II co-clinical trial of arsenic trioxide in relapsed small cell lung cancer

**DOI:** 10.1186/s12967-016-0861-5

**Published:** 2016-05-03

**Authors:** Taofeek K. Owonikoko, Guojing Zhang, Hyun S. Kim, Renea M. Stinson, Rabih Bechara, Chao Zhang, Zhengjia Chen, Nabil F. Saba, Suchita Pakkala, Rathi Pillai, Xingming Deng, Shi-Yong Sun, Michael R. Rossi, Gabriel L. Sica, Suresh S. Ramalingam, Fadlo R. Khuri

**Affiliations:** Department of Hematology & Medical Oncology, Emory University School of Medicine, Winship Cancer Institute, 1365C Clifton Road, NE, Suite C3080, Atlanta, GA 30322 USA; Department of Radiology, Division of Interventional Radiology, Emory University School of Medicine, Winship Cancer Institute, Atlanta, GA 30322 USA; Winship Cancer Institute, Atlanta, GA 30322 USA; Department of Medicine, Division of Interventional Pulmonology, Winship Cancer Institute, Atlanta, GA 30322 USA; Department of Biostatistics, Rollins School of Public Health and Biostatistics Shared Resource, Winship Cancer Institute, Atlanta, GA 30322 USA; Department of Radiation Oncology, Winship Cancer Institute, Atlanta, GA 30322 USA; Department of Pathology, Winship Cancer Institute, Atlanta, GA 30322 USA

**Keywords:** Small cell lung cancer, Arsenic trioxide, Clinical trial, Ex vivo, Patient-derived xenograft, Efficacy, Survival

## Abstract

**Background:**

SCLC has limited treatment options and inadequate preclinical models. Promising activity of arsenic trioxide (ASO) recorded in conventional preclinical models of SCLC supported the clinical evaluation of ASO in patients. We assessed the efficacy of ASO in relapsed SCLC patients and in corresponding patient-derived xenografts (PDX).

**Methods:**

Single arm, Simon 2-stage, phase II trial to enroll patients with relapsed SCLC who have failed at least one line of therapy. ASO was administered as an intravenous infusion over 1–2 h daily for 4 days in week 1 and for 2 days in weeks 2–6 of an 8-week cycle. Treatment continued until disease progression. Pretreatment tumor biopsy was employed for PDX generation through direct implantation into subcutaneous pockets of SCID mice without in vitro manipulation and serially propagated for five generations. Ex vivo efficacy of cisplatin (3 mg/kg i.p. weekly) and ASO (3.75 mg/kg i.p. every other day) was tested in PDX representative of platinum sensitive and platinum refractory SCLC.

**Results:**

The best response in 17 evaluable patients was stable disease in 2 (12 %), progressive disease in 15 (88 %) patients and median time-to-progression of seven (range 1–7) weeks. PDX was successfully grown in 5 of 9 (56 %) transplanted biopsy samples. Serially-propagated PDXs preserved characteristic small cell histology and genomic stability confirmed by immunohistochemistry, short tandem repeat (STR) profiling and targeted sequencing. ASO showed in vitro cytotoxicity but lacked in vivo efficacy against SCLC PDX tumor growth.

**Conclusions:**

Cisplatin inhibited growth of PDX derived from platinum-sensitive SCLC but was ineffective against PDX from platinum-refractory SCLC. Strong concordance between clinical and ex vivo effects of ASO and cisplatin in SCLC supports the use of PDX models to prescreen promising anticancer agents prior to clinical testing in SCLC patients.

*Trial Registration* The study was registered at http://www.clinicaltrials.gov (NCT01470248)

**Electronic supplementary material:**

The online version of this article (doi:10.1186/s12967-016-0861-5) contains supplementary material, which is available to authorized users.

## Background

Small cell lung cancer (SCLC) constitutes approximately 15 % of all cases of lung cancer, which translates to 30,000 new cases in the US and almost 200,000 new cases worldwide each year [1, 2]. While it is a very chemosensitive disease in the frontline setting, the outcome is dismal with median survival of only 10 months and 2-year survival rate as low as 2.2 % [2, 3]. Relapsed SCLC is much less responsive to chemotherapy [3–5]. The underlying mechanism responsible for the high rate of relapse and the poor outcome for relapsed SCLC is poorly understood but progressive outgrowth of resistant clones selected by the initial chemotherapy regimen is one potential reason. Preclinical data in SCLC cell lines revealed the presence of a rare sub-clone with a stem-cell phenotype characterized by surface receptor expression of CD44, CD133, multi-drug resistance gene (MDR1) and urokinase plasminogen activator receptor (uPAR) [6–8]. This cell population frequently exhibits primary resistance to frontline chemotherapy agents including cisplatin and etoposide [7, 8]. Furthermore, these cells possess a high capacity for self-renewal, differentiation into adult cancer cells and increased tumorigenesis in clonogenic assays.

Targeting this population of cells with differentiating agents such as arsenic trioxide (ASO) is expected to confer clinical benefit especially in relapsed SCLC where prior therapies could enrich for this sub-clone of resistant undifferentiated cells. Indeed, ASO was shown in several preclinical studies to induce potent in vitro cytotoxicity against SCLC through the activation of both necrotic and apoptotic cell death pathways in SCLC cell lines [9–11]. It also achieved significant tumor growth delay in standard subcutaneous xenograft models derived from traditional SCLC cell lines [10–12].

Based on this preclinical data, we designed a phase II clinical trial to test the efficacy of ASO as salvage therapy for relapsed SCLC. Because of the repeated failure to translate preclinical signal into clinical activity in this disease, we also tested the efficacy of ASO ex vivo using patient-derived xenografts (PDX) established using tumor biopsy samples collected from patients enrolled in the study.

## Methods

The primary objective of the study was to determine the objective response rate (ORR) associated with ASO as treatment for relapsed SCLC. Other objectives were to determine the progression free survival and overall survival associated with single agent ASO in refractory SCLC and to correlate the clinical efficacy observed in patients with the activity in corresponding PDXs generated using pretreatment tumor biopsies obtained from patients enrolled on study. ASO was generously provided by Teva Pharmaceuticals, (North Wales, PA 19454 US). All patients were enrolled through the thoracic medical oncology clinic of the Winship Cancer Institute of Emory University. The study was conducted under a prospective clinical trial protocol approved by the Emory University Institutional Review Board (IRB00024810). Study conduct was in compliance with all ethical standards and good clinical practice. All study participants provided written informed consent prior to undergoing any protocol-related procedures. The study was registered at http://www.clinicaltrials.gov (NCT01470248).

### Eligibility

Adult patients 18 years or older with a pathologic diagnosis of SCLC and who progressed following at least one line of standard treatment were eligible for the study. There was no limit to the number of prior therapies. Other salient requirements included measurable disease by RECIST criteria, ECOG performance status ≤2, and normal organ and marrow function: absolute neutrophil count >1500/mL, platelets >100,000/mL, total bilirubin ≤1.5 X ULN, AST/ALT <2.5 X ULN, creatinine ≤1.5 X ULN or creatinine clearance >40 mL/min/1.73 m^2^. Due to the potential for cardiac toxicity with ASO, patients with history of QTc prolongation syndrome or any other cardiac conduction abnormality evidenced by abnormal baseline EKG (QTc ≤450 and ≤470 in males and females, respectively) or who required treatment with medication with potential to prolong the QT interval or induce Torsade’s de Pointes were excluded. Other pertinent exclusion factors included uncontrolled symptomatic brain metastases or intercurrent illness that would limit compliance with study requirements.

### Patient selection and treatment administration

Patients were entered into the study by competitive enrollment in the order in which they presented for the study. Patients started treatment within 2 weeks of registration and confirmation of eligibility. ASO was administered intravenously over 1–2 h as a loading dose of 0.32 mg/kg/day for 4 days in week 1 (days 1, 2, 3, 4) followed by 0.25 mg/kg/day twice per week (two consecutive days) for 5 weeks and then a 2 week rest period prior to the initiation of a new cycle for a schedule of 8 weeks per cycle. This dose and schedule of ASO were previously established to be safe in a phase II study in advanced melanoma patients [13]. Patients who experienced any delay in treatment during the cycle were not allowed to make up the missed doses, such that no cycle was longer than 8 weeks and the required 2-week rest period was observed prior to the beginning of a new cycle. Treatment was administered on an outpatient basis and continued for a maximum of six cycles not to exceed 85 daily infusions of ASO in line with the prescribing information. Restaging scan by cross sectional imaging was obtained prior to the beginning of a new cycle approximately every 8 weeks. Response assessment was performed according to the RECIST 1.0 criteria at the end of each cycle approximately every 8 weeks. Patients who completed or discontinued treatment were followed for survival at intervals of approximately 3 months until study closure or patient death, whichever occurred first.

### Ex vivo and in vitro efficacy testing using cell line and patient-derived xenograft

Short-term in vitro growth inhibition by cisplatin and ASO of TKO-002 cell line derived from a biopsy sample of one of the enrolled patients was assessed using MTS [(3-(4,5-dimethylthiazol-2-yl)-5-(3-carboxymethoxyphenyl)-2-(4-sulfophenyl)-2H-tetrazolium)/phenazine methosulfate (PMS)] colorimetric assay (Promega, Madison, WI) as we previously described [14]. Briefly, exponentially growing TKO-002 cells cultured in 96-well cell culture plates were treated by continuous exposure to vehicle or increasing concentrations of ASO (0.2–24 μM) and cisplatin (2–216 μM). In a separate experiment, fixed concentrations of ASO (6 μM) and cisplatin (2.5 and 10 μM) were tested singly and in combination. The inhibitory concentration (IC_50_) and percent growth inhibition by fixed doses of the cytotoxic agents was calculated using GraphPad Prism software (GraphPad Software, Inc. La Jolla, CA).

Ex vivo efficacy testing was conducted under an animal protocol approved by the Emory University Institutional Animal Care and Use Committee (IACUC) and in accordance with guidelines for the humane treatment of laboratory animals. Pretreatment tumor biopsy was collected from consenting patients prior to initiation of treatment with ASO. Tumor samples were obtained by image-guided core needle biopsy or by bronchoscopy in patients with centrally located tumor. Tissue specimens were collected under sterile condition in antibiotic-containing RPMI medium and immediately transplanted as 2–3 mm^3^ pieces into subcutaneous pockets on each side of the lower back of two 6-week old female NOD SCID mice (Harlan Laboratories, Inc. Indianapolis, IN US) [15]. Tumors from the first generation of mice were harvested after reaching an approximate size of 1.5 cm in diameter and directly re-implanted into a new generation of five 6-week old female athymic nude mice (Harlan Laboratories, Inc. Indianapolis, IN US) for up to five generations. Remnant tumor samples (suspended in 10 % DMSO plus 10 % FBS containing medium) were cryopreserved in liquid nitrogen and kept as a live bank for future use. Representative samples from harvested tumors were confirmed as human SCLC using standard H&E and immunohistochemistry staining with antibodies against CD56 (NCMA1), synaptophysin and chromogranin. In addition, integrity, relatedness and human origin of the PDX were confirmed using short tandem repeat (STR) profiling. The profiles of ten core STR markers (TH01, D21S11, D5S818, D13S317, D7S820, D16S539, CSF1PO, AMEL, vWA, and TPOX) were tested to establish the unique profile of each PDX and to show the relatedness of different generations of each PDX to one another.

Anti-tumor efficacy of ASO was evaluated in two PDX models representative of platinum sensitive (TKO-005) and platinum refractory (TKO-002) SCLC. Briefly, actively growing subcutaneous PDX tumor was harvested and immediately implanted into cohorts of nu/nu mice. Once the tumor volume reached an average size of 100 mm^3^, PDX-bearing mice were matched for tumor size and treated in groups of 3–6 animals with vehicle (0.9 % NaCl i.p. weekly), cisplatin (3 mg/kg i.p. weekly) or ASO (3.75 mg/kg i.p. every other day). In a separate experiment we attempted to demonstrate the synergistic efficacy of the combination of ASO (7.5 mg/kg i.p. daily) and cisplatin (3 mg/kg i.p. weekly) as previously reported using conventional models of SCLC [10]. Animal weight and tumor growth were measured twice weekly. Tumor volume was calculated using external caliper measurement by the formula: $0.5 \times ( {length \times ( {width})^{2} } )$. Animals were euthanized and harvested tumors were weighed at the end of treatment.

### Targeted DNA sequencing

SNaPshot multiplex sequencing technique was employed to identify *TP53* gene mutations and other frequently mutated oncogenes in lung cancer including AKT1 (c.49G>A), BRAF (c.1397G>T, c.1406G>A/C/T, c.1789C>G, c.1799T>A), EGFR (c.2156G>A/C, c.2369C>T, c.2573T>G, c.2582T>A, exon.19.del, exon.20.ins), ERBB2 (ins.A775/exon.20.ins), KRAS (c.181C>A/G, c.182A>C/G/T, c.183A>C/T, c.34G>A/C/T, c.35G>A/C/T, c.37G>A/C/T, c.38G>A/C/T, c.180.181TC>CA), MEK1 (c.167A>C, c.171G>T, c.199G>A), NRAS (c.181C>A/G, c.182A>C/G/T) and PIK3CA (c.1624G>A/C, c.1633G>A/C,c.3140A>G/T). Harvested tumor samples from the PDX were employed for targeted sequencing. Sample preparation and genetic mutation identification followed previously described methodologies [16].

### Statistical analysis

The clinical study was designed using a MiniMax design to test the hypothesis that ASO will achieve an overall response rate (ORR) of 20 % in relapsed SCLC. The study had 90 % power at an alpha level of 10 % to distinguish a promising RR of 20 % if the agent is active versus a 5 % ORR if inactive. In the first stage of the study, ≥1 of 18 eligible patients with an objective response by RECIST criteria was required in order to enroll 14 additional patients to the second stage of the study for a total accrual goal of 32 patients. At the end of stage II accrual, ≤4 of the 32 patients must have achieved an objective response for ASO to be deemed active in the relapsed SCLC patient population. An alternative endpoint of clinical benefit rate (CBR i.e., sum of CR, PR or SD) was also pre-specified in the event that the study failed to meet its ORR endpoint either at the end of stage I accrual or at final analysis. A CBR of 40 % would be considered sufficient justification for further evaluation of ASO in SCLC. All patients who received any amount of the study drug were evaluable for toxicity using the CTCAE version 4 criteria. Receipt of at least 75 % of the planned dose intensity in cycle 1 was required for a patient to be deemed evaluable for efficacy using RECIST criteria 1.1. Differences at the end of treatment in mean tumor volume and mean harvested tumor weights between animal groups treated with vehicle, ASO or cisplatin was compared in a pair wise fashion using one-sided Student t test.

## Results

### Patient characteristics

We enrolled 20 eligible patients including 13 males and seven females. Nineteen patients initiated treatment as prescribed by protocol. The median age was 63 years. Eleven patients had platinum sensitive SCLC (defined as disease progression >90 days from the end of frontline therapy) and eight patients had known brain metastasis at the time of enrollment on study. The full details of patient and tumor characteristics as well as prior therapies are provided in Table 1.

**Table 1:** Patient characteristics and summary of treatment efficacy

Variable	Level	N = 20	%
Gender	F	7	35.0
	M	13	65.0
Race	White	16	80.0
	Black	4	20.0
Age (years)	Mean	63.40 (10.67)	
	Median	63 (48–84)	
Age (years)	<65	10	50.0
	≥65	10	50.0
ECOG performance status	0	2	10
	1	13	65
	2	5	25
No of prior therapies	1^a^	6	30
	2^b^	6	30
	3^c^	7	35
	4^d^	1	5
Best response	PD	15	88.2
	SD	2	11.8
Time to progression (TTP)	Mean	6.26 (3.9)	
	Median	7 (1–17)	

^a^Platinum/etoposide; platinum etoposide/XRT, platinum/etoposide/GDC-049

^b^Topotecan, oral etoposide, platinum/irinotecan, platinum/etoposide, topotecan/aflibercept; gemcitabine, paclitaxel

^c^Platinum etoposide, platinum/irinotecan, topotecan, irinotecan

^d^Irinotecan

### Adverse events

Most patients experienced grade 1 and 2 adverse events. The most frequent grade 3 or 4 adverse events are detailed in Table 2 and there were no grade 5 adverse events recorded on study.

**Table 2:** List and frequency of grade ≥3 adverse events

Adverse event	N (%)	Grade
Dyspnea	1 (4.5)	3
Anemia	1 (4.5)	3
Back pain	1 (4.5)	3
Elevated creatinine	1 (4.5)	3
Facial edema around eyes	1 (4.5)	3
Generalized weakness	1 (4.5)	3
Hyperbilirubinemia	2 (9)	3
Hyperglycemia	1 (4.5)	3
Hypoalbuminemia	2 (9)	3
Hypocalcemia	1 (4.5)	3
Hyponatremia	1 (4.5)	3
Hypophosphatemia	1 (4.5)	3
Increased alkaline phosphatase	1 (4.5)	3
Increased lipase	1 (4.5)	3
Leukopenia	2 (9)	3
Neutrophil count decreased	1 (4.5)	3
Pleural effusion	1 (4.5)	3
Hyperbilirubinemia	1 (4.5)	4

### Clinical efficacy

Seventeen patients were evaluable for efficacy as defined by the protocol. There were no complete or partial responses. The best outcome was stable disease in two patients and progressive disease in 15 patients. The median time to progression was 7 weeks (1–17 weeks) while the median overall survival was 4.5 months (2–7 months); 6- and 12-month survival rates were 30.0 % (12.3, 50.1 %) and 10.0 % (1.7, 27.2 %) respectively. Only one of 20 enrolled patients was still alive at the time of data analysis. There was no significant association between clinical efficacy and patient characteristics. However, a non-significant trend was observed toward higher risk of death in patients older than 65 years of age (HR 2.85; 95 % CI 0.95–8.55; p = 0.061).

### Patient-derived xenografts (PDX)

Nine patients underwent pretreatment tumor biopsy for the purpose of generating PDX. Implanted tissue biopsies and corresponding PDX and cell lines were serially numbered as TKO-001 through TKO-009. We successfully generated PDXs from 5 of 9 patients for a take rate of 56 %. The mean and median time to PDX development post initial direct implant from the patient biopsy were 172 (±114) days and 135 days (range 49–335 days), respectively. PDXs were eventually propagated across five generations of mice. There was a progressive decrease in the interval of time from implant to tumor development with serial passage.

### PDX purity and relatedness

**Fig. 1:**
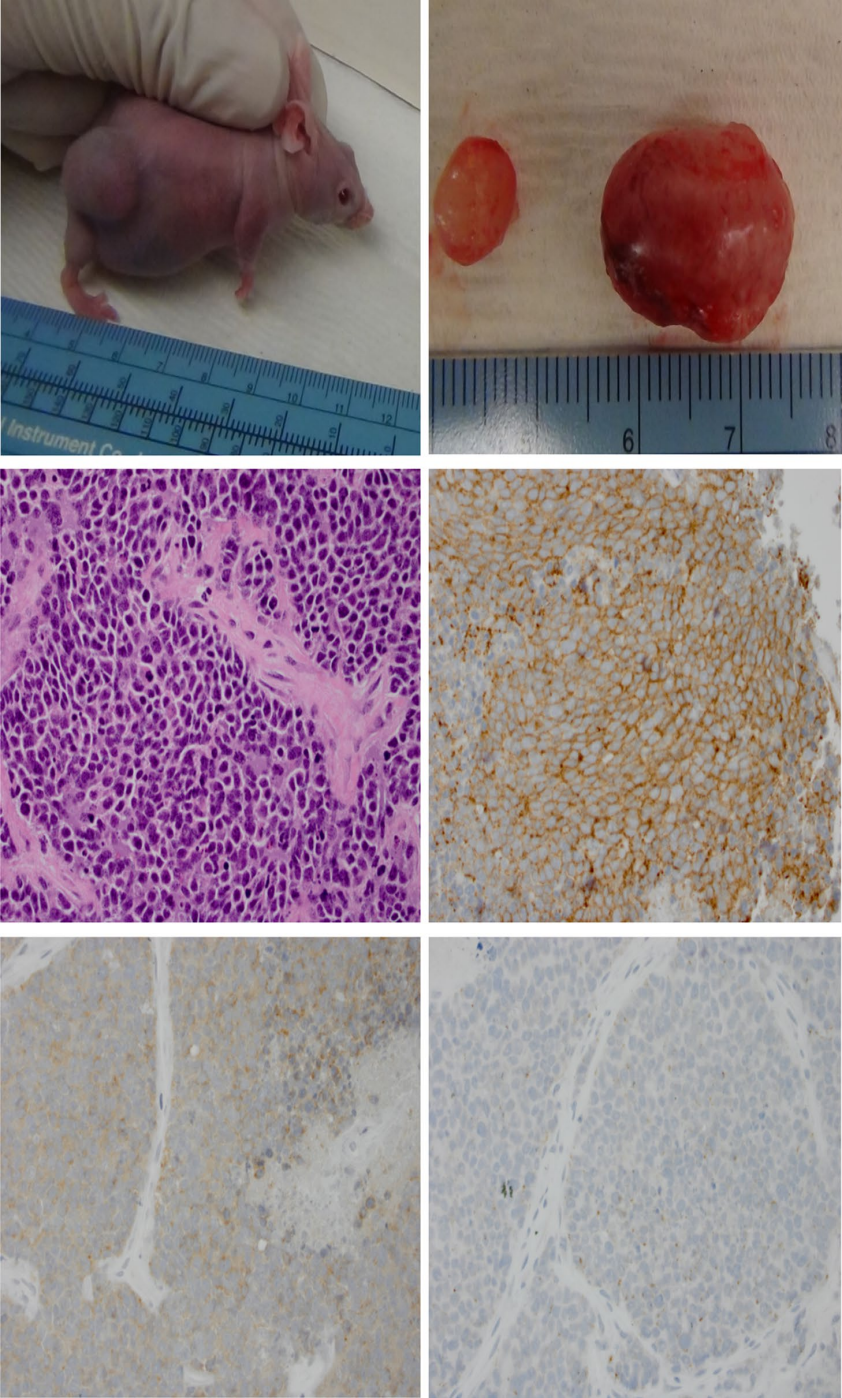
Subcutaneous growth of patient-derived xenograft in a SCID mouse host just prior to euthanasia. Harvested tumor from bilateral subcutaneous pockets; 3 × 3 mm sized sections were immediately propagated to the next generation of mice through implantation into subcutaneous pockets over the hind legs of the mice without in vitro manipulation (*top panel*). Histopathologic confirmation of small cell lung carcinoma histology by hematoxylin and eosin stain (X400) and immunohistochemistry for neuroendocrine differentiation showing intense diffusely positive staining for CD56 (*middle panel*), moderately intense staining for synaptophysin and focal areas of weakly positive chromogranin A staining (*bottom panel*)

**Fig. 2:**
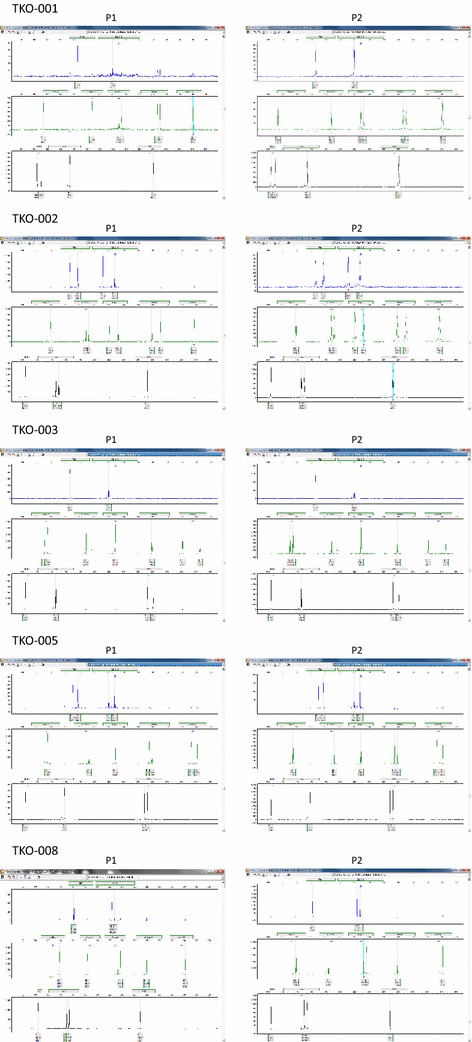
Electropherogram showing unique profile of each of five different PDX models generated from SCLC. Note the identical STR profile of tumor samples harvested from animals bearing first generation (*passage 1*) and second generation (*passage 2*) PDX

The PDX preserved the characteristic histopathologic features of human SCLC with serial passage (Fig. 1; Additional file 1: Fig. S1). PDX purity across the first two generations for each of the five PDXs was authenticated using STR profiling (Fig. 2; Table 3). Due to variable rate of growths of the PDX, we used samples from the first two generations of each PDX for STR profiling. Furthermore, targeted sequencing of the harvested PDX using a standard clinical NexGen sequencing platform employed at our institution recapitulated the genetic characteristics of human SCLC with universal alterations in *TP53* gene. There was also relative stability of the genomic alterations across tumor generations (Table 4). Additionally, re-implantation of representative tumor samples that had been stored in liquid nitrogen for more than 2 years led to successful tumor regrowth in mice. Detailed clinical information of nine patients who underwent biopsy for PDX generation and outcomes are presented in Table 5.

**Table 3:** STR profiling of PDX tumor samples

PDX	TH01		D21S11		D5S818		D13S317		D7S820		D16S539		CSF1PO		AMEL		vWA		TPOX	
TKO-001_P1	7	7	29	29	7	7	11	11	10	11	11	12	12	12	X	Y	18	18	11	11
TKO-001_P2	7	7	29	29	7	7	11	11	10	11	11	12	12	12	X	Y	18	18	11	11
TKO-002_P1	7	9.3	27	31	13	13	11	12	8	11	9	12	11	11	X	X	16	17	9	9
TKO-002_P2	7	9.3	27	31	13	13	11	12	8	11	9	12	11	11	X	X	16	17	9	9
TKO-003_P1	7	7	29	29	11	12	11	11	10	10	9	9	7	13	X	X	16	16	9	11
TKO-003_P2	7	7	29	29	11	12	11	11	10	10	9	9	7	13	X	X	16	16	9	11
TKO-005_P1	8	9.3	29	31	12	12	12	12	10	10	8	9	10	12	X	X	19	19	8	9
TKO-005_P2	8	9.3	29	31	12	12	12	12	10	10	8	9	10	12	X	X	19	19	8	9
TKO-008_P2	6	6	30	31	13	13	10	10	11	12	9	9	12	12	X	X	17	18	8	8
TKO-008_P1	6	6	30	31	13	13	10	10	11	12	9	9	12	12	X	X	17	18	8	8

**Table 4:** Specific genetic alterations in TP53 gene detected in the five SCLC PDXs

PDX	TP53 alteration	COSMIC ID#	Remarks
TKO-001	c.422G>A[p.Cys141Tyr]	COSM131470	Missense mutation
TKO-002	c.488A>G[p.Tyr163Cys]	COSM10808	Listed in COSMIC and dbSNP (rs148924904), evidence in COSMIC for being somatic
TKO-003	c.913A>T[p.Lys305Ter]	COSM43773	Stop (Ter) Hemizygous, deletion of one copy
TKO-005	c.488A>G[p.Tyr163Cys]	COSM10808	Listed in COSMIC and dbSNP (rs148924904), evidence in COSMIC for being somatic
TKO-008	c.892G>T[p.Glu298Ter]	COSM10710	Stop (Ter) 80 % of reads

**Table 5:** Characterisitics of patients and corresponding PDXs

	Clinical history	Prior treatment	Days to initial tumor growth	Cell line generation
TKO-001	PDX from biopsy specimens of hepatic metastasis in a 72-year-old hispanic male	Surgical resection cisplatin and etoposide with good response Platinum sensitive recurrence retreated with platinum/etoposide salvage therapy with irinotecan	335	No
TKO-002	PDX from biopsy of hepatic metastasis from a female white patient	Frontline carboplatin/etoposide with tumor response after two cycles and progression after the fourth cycle Single agent etoposide Topotecan	49	Yes
TKO-003	PDX from bronchoscopic biopsy samples obtained from a paratracheal mass and subcarinal lymph node metastasis in a 71-year-old African American male	Initial diagnosis of limited stage SCLC treated with combined radiation and cisplatin/etoposide. Following recurrence 2 years later of his platinum sensitive disease, he was retreated with carboplatin/etoposide and more recently was treated with topotecan prior to study enrollment	135	No
TKO-004			No growth	No
TKO-005	PDX developed using bronchoscopic biopsy samples obtained from a left lower lung nodule in a 50 year old African American male	Previously treated with combined radiation and cisplatin/etoposide for limited stage SCLC. Patient had disease progression more than 6 months after completion of chemoradiation and had no other systemic therapy prior to coming on this clinical trial when the biopsy was obtained to generate the PDX	103	No
TKO-006			No growth	No
TKO-007			No growth	No
TKO-008	PDX generated using lung biopsy from a 76-year-old male Caucasian patient	Previously treated with carboplatin/etoposide for six cycles. He subsequently received paclitaxel for progressive disease that occurred 4 months after completing frontline therapy	239	No
TKO-009			No growth	No

### In vitro and ex vivo efficacy of ASO and cisplatin in PDXs

**Fig. 3:**
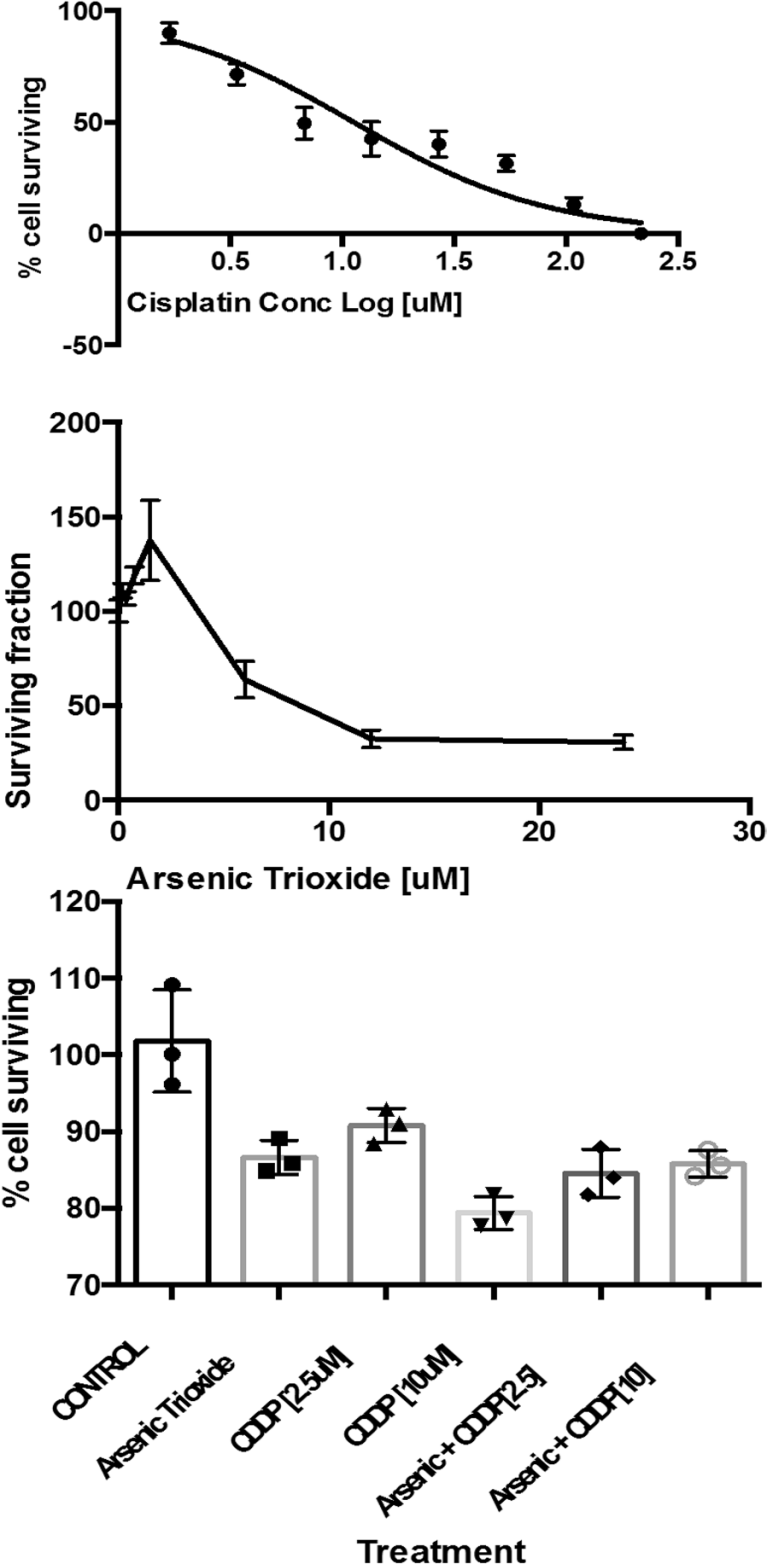
SCLC cell line derived directly from a tumor biopsy specimen employed for the generation of TKO-002 PDX was employed for in vitro cytotoxicity assessment. TKO-002 cells were seeded in 96-well plates and allowed to grow overnight. Exponentially growing cells were treated the next day with vehicle or serially increasing concentrations of cisplatin (2–216 μM) and ASO (0.2–24 μM). After 72 h of continuous drug exposure, cell numbers were estimated using MTS assay. IC_50_ concentration was estimated from the growth inhibition using GraphPad prism software. The IC_50_ concentration for cisplatin (*top panel*) and ASO (*middle panel*) was estimated at 11.25 and 3.08 μM, respectively. There was no demonstrable additive or synergistic effect of the combination of ASO (6 μM) and cisplatin (2.5 or 10 μM) over each agent alone against TKO-002 cells (*bottom panel*)

We were successful in establishing the optimal condition for exponential growth of cell lines obtained from patient biopsy for in vitro work from TKO-002 only. Both cisplatin and ASO demonstrated modest in vitro cytotoxicity against TKO-002 with IC_50_ concentrations of 11.25 and 3.08 μM respectively (Fig. 3, top and middle panel). The combination of ASO and cisplatin, however, did not show any additive or synergistic effect against TKO-002 cell line in vitro (Fig. 3, bottom panel).

**Fig. 4:**
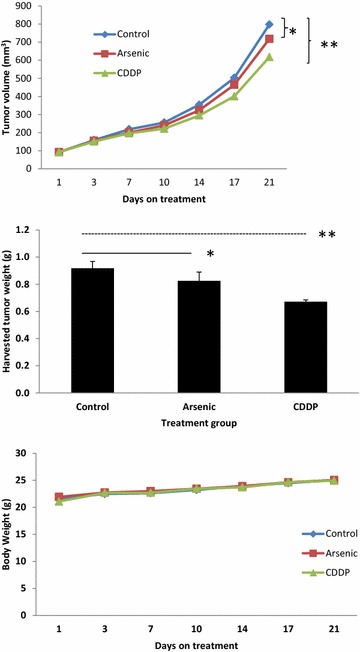
Efficacy of ASO (3.75 mg/kg i.p. every other day) and cisplatin (3 mg/kg i.p. weekly) was tested in TKO-002, a PDX model of platinum refractory SCLC. Tumor volume (mm^3^) and body weight of animals were measured at least twice weekly while on treatment. There was no significant tumor growth inhibition by ASO (*p = 0.48) or cisplatin (**p = 0.42) in comparison to vehicle-treated control animals at the end of the treatment period (*top*). There was also no significant difference in the harvested tumor weights from animals treated with ASO compared to control animals treated with vehicle (*p = 0.33) (*middle*). There was no significant increase in toxicity (measured by body weight of the animals) with active therapy in comparison to controls (*bottom*)

Ex vivo efficacy of ASO and cisplatin was assessed in two representative PDXs, TKO-002 and TKO-005, generated using biopsy samples from patients with platinum refractory and platinum sensitive relapsed SCLC, respectively. The PDX samples employed for ex vivo testing were the second generation PDX for each case. The source patients for the two PDXs were evaluable for efficacy assessment following treatment with ASO on the human clinical trial. PDX-bearing animals were treated with vehicle (no expected effect), cisplatin (to model platinum resistance or sensitivity) and ASO (to replicate clinical efficacy in the patients). Compared to vehicle-treated animals, there was no significant inhibition of TKO-002 PDX tumor growth by cisplatin (p = 0.42) or ASO (p = 0.48), and no significant difference in harvested tumor weights between vehicle and ASO-treated tumors (p = 0.33) at the end of treatment (Fig. 4). Of note, this PDX was generated from a patient with platinum refractory disease.

**Fig. 5:**
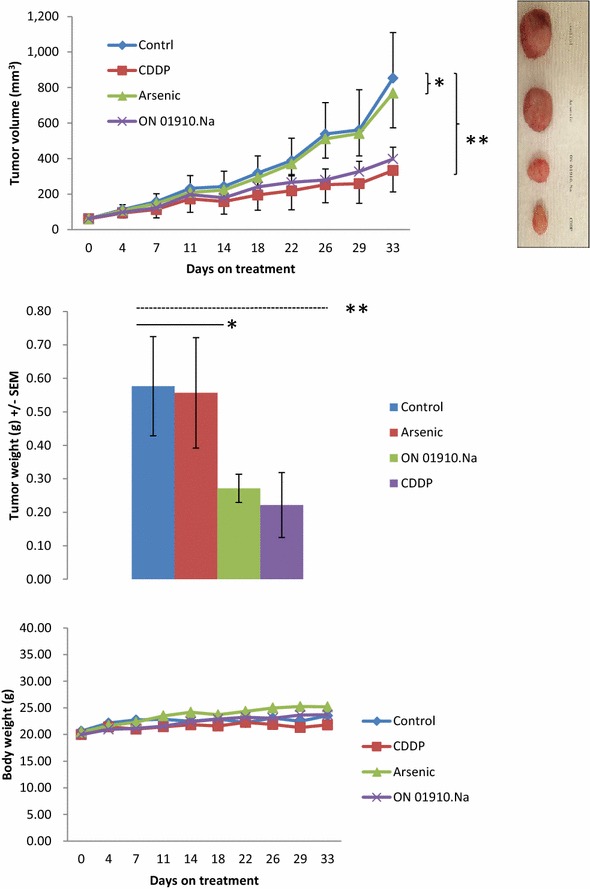
To assess the efficacy of ASO and cisplatin (CDDP) in TKO-005, a PDX model of platinum sensitive SCLC, animals were treated and monitored for tumor growth and body weight as described in Fig. 4. In addition, a matching group of tumor-bearing mice was treated with rigosertib (250 mg/kg i.p. daily). At the end of the treatment period, there was no significant reduction in tumor volume in animals treated with ASO (*p = 0.40) but a significant reduction was achieved with cisplatin (**p = 0.048) and a strong trend toward reduced tumor volume was noted with rigosertib (p = 0.058) in comparison to vehicle-treated control animals. Similarly, harvested tumor weights were significantly lower from animals treated with cisplatin (**p = 0.04) and rigosertib (p = 0.038) but not from animals treated with ASO (*p = 0.46) in comparison to control animals. There was no significant increase in toxicity as measured by body weight of the animals on active therapy in comparison to controls. Furthermore, rigosertib (ON-01910.Na) efficacy was comparable to cisplatin both in terms of growth inhibition (p = 0.24) and harvested tumor weights (p = 0.32) at the end of treatment

Contrarily, while ASO showed no significant efficacy in terms of tumor growth inhibition (p = 0.40) or harvested tumor weight (p = 0.46) against TKO-005 PDX, there was significant antitumor effect of cisplatin against this PDX, which was generated from a patient with platinum sensitive disease, as indicated by the significant reduction in harvested tumor weight (p = 0.04) with a trend in reduced tumor volume (p = 0.05) at the end of treatment (Fig. 5). In order to test whether the PDX model could predict efficacy of other agents beyond cisplatin, we also evaluated the in vivo efficacy of ON.01910.Na (rigosertib), a novel agent, targeting polo-like kinase 1 (PLK1), which was previously identified as a promising target in SCLC [17]. When tested along with cisplatin and ASO in this platinum-sensitive model, we observed that the efficacy of the PLK1 inhibitor to be comparable to cisplatin in this PDX model (Fig. 5).

**Fig. 6:**
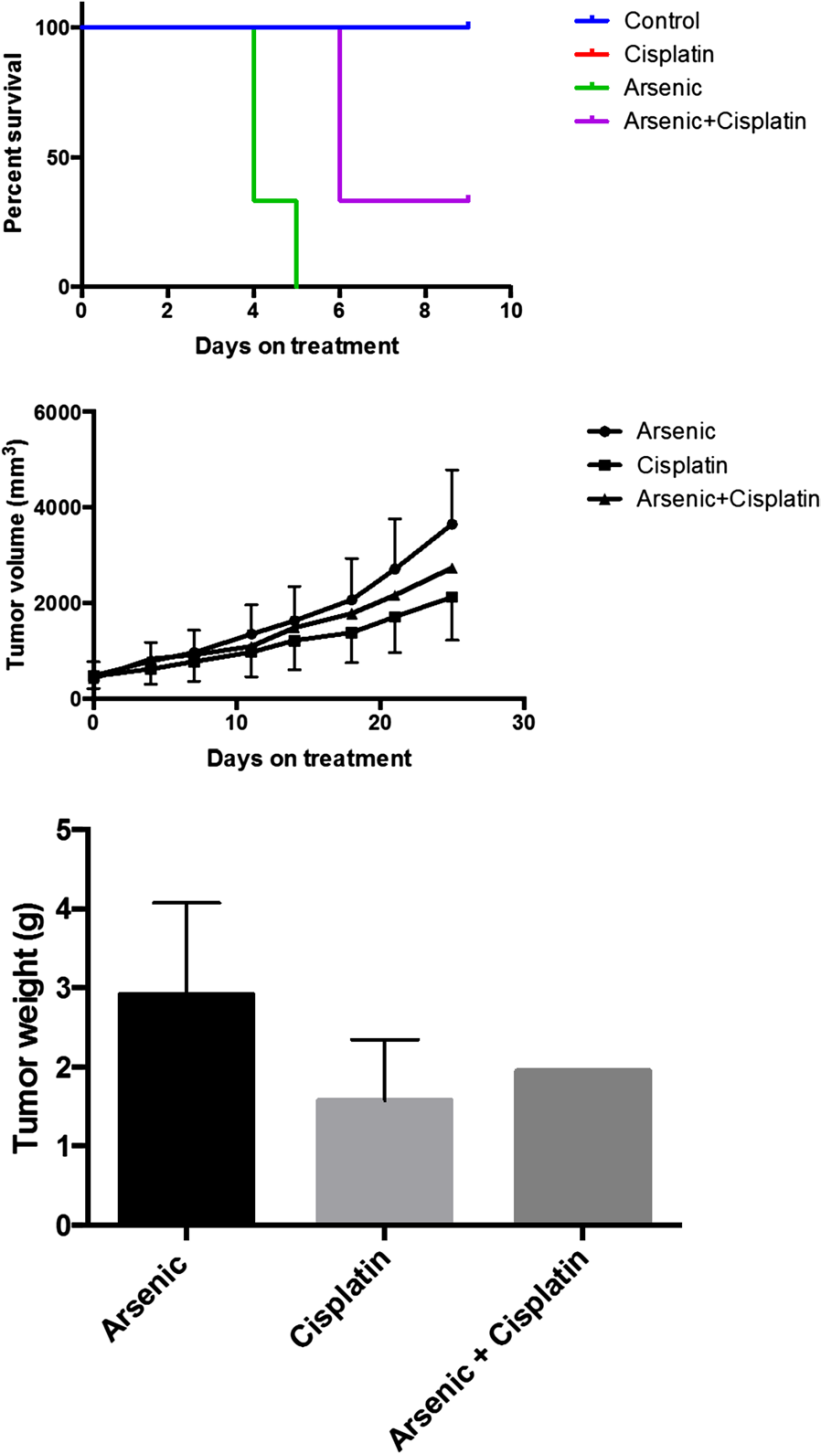
Efficacy of ASO and cisplatin (CDDP) singly and in combination was tested in TKO-002, a PDX model of platinum refractory SCLC. Kaplan–Meier survival curves for animal groups treated with vehicle, ASO (7.5 mg/kg i.p. daily), cisplatin (3 mg/kg i.p. weekly) and the combination of ASO plus cisplatin. There was significant toxicity with rapid death of mice treated with ASO alone or in combination with cisplatin (*top*). A reduced dose of ASO (3.75 mg/kg every other day) was better tolerated but showed negligible efficacy and failed to potentiate the minimal growth inhibition achieved by cisplatin (3 mg/kg i.p. weekly) in this PDX model derived from a patient with platinum resistant SCLC (*middle* and *bottom panel*)

The combination of ASO and cisplatin was shown to be synergistic in preclinical models of SCLC and other cancers [10, 18–20]. We attempted to replicate this observation in our SCLC PDX model in order to further explore whether this strategy holds sufficient promise to warrant future clinical testing. There was significant toxicity of single agent ASO and its combination with cisplatin (Fig. 6) at the dose (7.5 mg/kg i.p daily) previously reported to be effective in SCLC xenograft model [10]. The reduced dose (3.75 mg/kg i.p) was better tolerated but did not have any significant effect on tumor growth as a single agent and failed to potentiate cisplatin in this PDX model of SCLC (Fig. 6).

## Discussion

We report the outcome of a prospective co-clinical trial of ASO in relapsed SCLC patients and corresponding PDX models. ASO is an effective therapy in acute promyelocytic leukemia (APL) [21, 22] and has been shown to be active in several other cancer types [13]. Despite findings from preclinical studies of in vitro and in vivo activity of ASO against SCLC, which suggested a high potential for clinical benefit of this agent in SCLC, [10–12], we could not demonstrate any significant anticancer efficacy of ASO in relapsed SCLC patients treated as part of this co-clinical trial. PDX models are becoming increasingly recognized as superior to traditional xenograft tumor models in faithfully representing the behavior of cancer in patients. We therefore incorporated PDX generation as a component of this clinical trial. Our success rate in establishing PDXs of 56 % compared well to the reported experience of other groups using circulating tumor cells or similar tissue biopsy techniques as we employed in this study [23–25]. This co-clinical trial approach allowed us to further interrogate the possible reasons for the failure to observe meaningful clinical benefit in the clinical trial participants despite the prediction of clinical efficacy of ASO by experiments in SCLC cell lines and traditional xenografts [10–12]. We, however, observed a consistency in outcome between patients and the corresponding representative PDX models and in the TKO-002 SCLC cell line, which was generated from a platinum refractory patient. The estimated IC_50_ concentration of cisplatin against this line of 11.25 μM is several folds higher than the range of 2–6.5 μM that is the achievable mean plasma concentration of free unbound and total cisplatin in patients [26, 27]. This result supports the expectation of platinum resistance in this cell line. We were unable to generate stably proliferating cell line for in vitro replication of platinum sensitivity in TKO-005, which was obtained from a patient with platinum sensitive disease. The estimated IC_50_ for ASO in TKO-002 of 3.08 μM is within the range of 3–7 μM achievable in plasma of patients [21, 28, 29]; and is comparable to the IC_50_ concentrations in APL cell lines [28]. However, human PK study showed a rapid clearance of ASO from the plasma within hours suggesting that intermittent dosing is suboptimal for maintaining the required drug level in patients. Overall, our data provide a reasonable ground to contend that prior screening of ASO using a PDX model along with or in place of the standard cell-derived xenograft could have provided a better hint of the likely clinical result thereby informing a reconsideration or redesign of the clinical trial. Our data supports the conclusion that failure to demonstrate clinical benefit both in terms of response or durable disease control is most likely due to a true lack of activity of ASO in unselected relapsed SCLC patients.

Other possible explanations for the lack of benefit include the fact that we employed an intermittent schedule of drug administration over 2–4 days in order to minimize hematologic toxicity unlike in APL patients where ASO is administered as a daily continuous regimen. This dosing schedule was previously established as the safe dose in patients with solid malignancies [13] as opposed to the continuous dosing employed in patients with APL [28]. While this can be overcome by the ability of APL cell to trap ASO intracellularly, it is unknown whether other cancer cells possess the same capability [28]. Although the relative reduction in dose intensity in our study in comparison to the dose and schedule employed for APL patients, where the agent is particularly effective could be one of the factors contributing to the negative result of the clinical trial. However, treatment of PDX-bearing mice with the same dose and schedule of ASO as was shown in earlier studies to have promising efficacy in conventional xenograft models failed to inhibit tumor growth [10, 11]. This suggests that the lack of clinical efficacy reflects the inadequacy of the traditional xenograft model to accurately predict outcome in patients. Additionally, ASO as a differentiating agent is expected to have greater effect on the undifferentiated tumor compartment rather than the entire tumor bulk. The undifferentiated compartment represents a smaller fraction of the total tumor bulk in solid tumors as compared to APL. Nonetheless, SCLC tends to have a high proportion of undifferentiated cells. We confirmed this by testing for expression of CD133 and CD87, which are established markers of undifferentiated cells in our PDX samples, and observed high expression of both of these markers across multiple generations of PDX (Additional file 1: Fig. S1). It is plausible that ASO may be more efficacious when combined with a cytotoxic agent that targets the remaining differentiated tumor compartment. Consistent with this expectation, Zheng et al. showed in preclinical models using the H841 SCLC cell line xenograft that the combination of ASO and cisplatin is much more potent than either agent alone [10]. However, we were unable to replicate this synergistic interaction using our SCLC PDX model in part due to excessive toxicity of the combination of cisplatin and ASO at doses required for clinical activity.

The concordance of the limited clinical efficacy of ASO and the lack of antitumor efficacy in the PDX model may be agent specific and thus may not be generalizable to other agents with different mechanism of action. Moreover, inherent genetic heterogeneity in tumors may limit concordance of PDX and clinical outcome since PDXs are generated using biopsy samples obtained from limited sites of tumor involvement. It is anticipated that these reasonable grounds of skepticism will be further addressed in future co-clinical trials and with increased use of the PDX model as a screening platform. However, we have employed some of the PDX models generated from this co-clinical trial to screen other classes of anticancer compounds including rigosertib, an inhibitor of PLK-1, which was previously shown to have potent in vitro activity against SCLC [17] as well as BDA-366, a novel BH4-mimetic Bcl2 inhibitor, with which we observed significant antitumor effect [30]. Furthermore, translational studies conducted by other groups but not as part of a prospective co-clinical trial also showed that SCLC PDX models replicate clinical activity of standard chemotherapy agents [31–33] and showed greater concordance with clinical experience in comparison to results obtained from in vitro and cell line derived xenograft experiments [34]. While it may be premature at this point to generalize that PDX model should be the gold standard platform for preclinical screening of novel agents against SCLC, it is safe to suggest that PDX model should be a component of the systematic preclinical screening strategy for novel agents against SCLC in order to improve the chances of clinical success.

## Conclusions

We report a prospectively designed co-clinical trial of anticancer agent therapy in SCLC patients and PDX models. While ASO failed to demonstrate meaningful clinical activity, the PDX models generated in the context of this study provide great insight into optimal platform for preclinical testing of novel therapeutic strategies in SCLC.

## Additional file

10.1186/s12967-016-0861-5 We employed immunohistochemistry to assess the level of expression of CD133 (1:100 dilution; mouse monoclonal antibody; Cat# MAB4310, EMD Millipore Corporation, Billerica, MA USA) and CD87 (1:100 dilution; rabbit polyclonal antibody; Cat# PA5-15478, Invitrogen, Thermo Fisher Scientific, Rockford, IL 61105 USA) in representative tumor tissues harvested from the first three generations of the five PDXs. There was weak to moderate degree of expression as measured by immunoscore (product of staining intensity and percent cell staining) with comparable expression between the five SCLC PDXs and across the three generations of each PDX.

## Abbreviations

SCLC: small cell lung cancer
ASO: arsenic trioxide
PDX: patient-derived xenograft
ULN: upper limit of normal
SD: stable disease
PR: partial response
CBR: clinical benefit rate
