# Supplementary figures and images for: Patient-derived xenografts faithfully replicated clinical outcome in a phase II co-clinical trial of arsenic trioxide in relapsed small cell lung cancer
Source: J Transl Med. 2016 May 3;14:111. doi: 10.1186/s12967-016-0861-5 (PMC4855771; doi:10.1186/s12967-016-0861-5)

## Slide 1
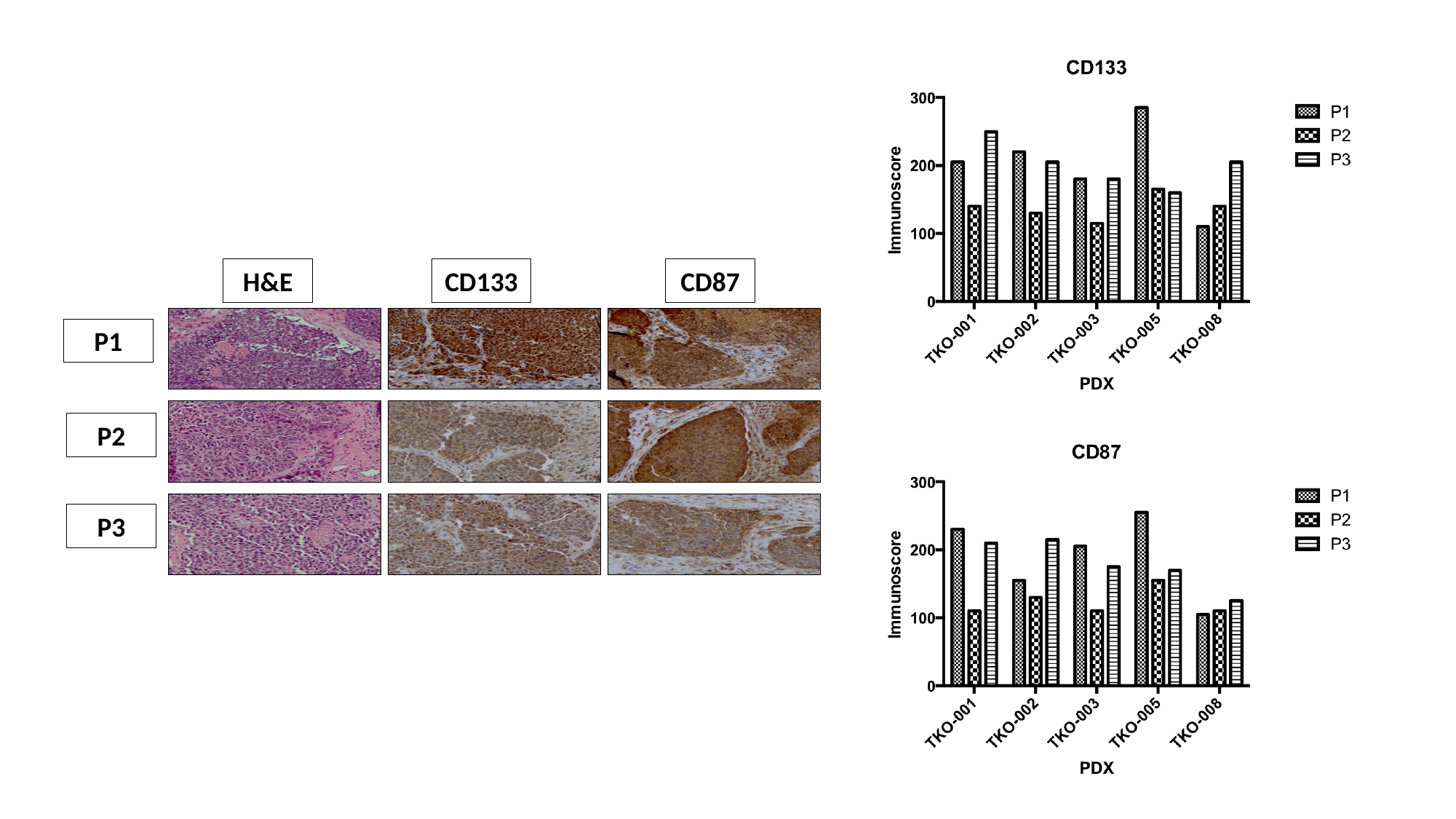

CD133
CD87
H&E
P1
P2
P3

Supplement: Supplementary file 1 — 10.1186/s12967-016-0861-5 We employed immunohistochemistry to assess the level of expression of CD133 (1:100 dilution; mouse monoclonal antibody; Cat# MAB4310, EMD Millipore Corporation, Billerica, MA USA) and CD87 (1:100 dilution; rabbit polyclonal antibody; Cat# PA5-15478, Invitrogen, Thermo Fisher Scientific, Rockford, IL 61105 USA) in representative tumor tissues harvested from the first three generations of the five PDXs. There was weak to moderate degree of expression as measured by immunoscore (product of staining intensity and percent cell staining) with comparable expression between the five SCLC PDXs and across the three generations of each PDX. [file 12967_2016_861_MOESM1_ESM.pptx]
